# Tick-Borne Pathogens in Companion Animals and Zoonotic Risk in Portugal: A One Health Surveillance Approach

**DOI:** 10.3390/microorganisms13081774

**Published:** 2025-07-30

**Authors:** Rita Calouro, Telma de Sousa, Sónia Saraiva, Diana Fernandes, Ana V. Mourão, Gilberto Igrejas, José Eduardo Pereira, Patrícia Poeta

**Affiliations:** 1 Veterinary and Animal Research Centre (CECAV), University of Trás-os-Montes and Alto Douro, 5000-801 Vila Real, Portugal; ritasousacalouro@gmail.com (R.C.); soniasaraiva@utad.pt (S.S.); jeduardo@utad.pt (J.E.P.); 2 MicroART-Antibiotic Resistance Team, Department of Veterinary Sciences, University of Trás-os-Montes and Alto Douro, 5000-801 Vila Real, Portugal; telmaslsousa@hotmail.com (T.d.S.); dianaivfernandes@gmail.com (D.F.); vanessamourao123@gmail.com (A.V.M.); 3 Associate Laboratory for Animal and Veterinary Science (AL4AnimalS), University of Trás-os-Montes and Alto Douro, 5000-801 Vila Real, Portugal; 4 Department of Genetics and Biotechnology, University of Trás-os-Montes and Alto Douro, 5000-801 Vila Real, Portugal; 5 Functional Genomics and Proteomics Unit, University of Trás-os-Montes and Alto Douro, 5000-801 Vila Real, Portugal; 6 Associated Laboratory for Green Chemistry, University NOVA of Lisbon, 1099-085 Caparica, Portugal

**Keywords:** arbovirus, emergence, *Ixodidae*, hemoparasite, public health, vector

## Abstract

This study aimed to assess the emergence and/or re-emergence of Tick-borne Diseases (TBD) in Portugal by linking the hemoparasite burden in companion animals to vector-borne disease dynamics through a One Health approach. Between 2015 and 2024, 1169 clinically suspected animals with hemoparasite infections, treated at the Hospital Veterinário de Santarém (HVS), underwent serological confirmation for *Rickettsia conorii*, *Babesia canis*, *Ehrlichia* spp., and *Haemobartonella* spp. A total of 3791 serological tests (3.2 tests per animal) were performed and 437 animals tested positive for at least one of the four hemoparasites under investigation. From 2020 to 2024, tests nearly tripled from 894 to 2883, raising positive cases and prevalence from 29.5% to 39.9%, especially for rickettsiosis and hemobartonellosis, indicating an increased circulation of their vectors. A national vector surveillance initiative identified *Hyalomma* spp., *Rhipicephalus sanguineus*, *Ixodes ricinus*, and *Dermacentor* sp. as primary tick vectors in Portugal for the hemoparasites mentioned above and for other agents like arbovirus, such as Crimean-Congo Hemorrhagic Fever Virus (CCHFV) and tick-borne encephalitis virus (TBEV). This study found that the vectors responsible for transmitting hemoparasitosis, given the high number of serologically positive cases detected in the HVS, represent an increasing risk for TBD. These findings highlight the relevance of companion animal monitoring as an early-warning component within a One Health surveillance approach.

## 1. Introduction

Vector-borne diseases (VBDs) represent a major public health challenge, especially in the context of emerging zoonoses. Among the main vectors involved in the transmission of pathogens to humans and companion animals are ticks, whose presence is associated with the spread of various hemoparasites with zoonotic potential [1,2].

Companion animals, particularly dogs and cats, play a central role in the epidemiological surveillance of tick-borne agents, acting as sentinel hosts that reflect the environmental risks shared with humans [3]. The identification of hemoparasites in dogs and cats has been documented in several European countries, including Portugal, with different levels of prevalence [4,5,6].

The zoonotic relevance of these agents has been recognized, although human infection with some of them is rare or underdiagnosed. However, human exposure to infected ticks shared with companion animals represents a real risk, particularly in rural or peri-urban environments where the proximity between humans, animals and vectors is high [7]. Thus, active surveillance of hemoparasite infection in dogs and cats can help to anticipate possible zoonotic threats by providing relevant data on the local circulation of pathogens [8]. While infected ticks serve as the principal transmission vectors for these hemoparasites, their emergence and dissemination are influenced by multiple factors, including climate change, human movement, trade, land use, and host–vector interactions [9,10,11,12,13]. Climate shifts have extended the geographic range and activity period of several tick species, including *Hyalomma marginatum*, *Ixodes ricinus*, and *Rhipicephalus sanguineus* [14,15]. Since the 1970s, the average annual temperature in mainland Portugal has risen by around 0.3 °C per decade. Among the hottest years on record are 2017 and 2023, the latter being the second hottest year since 1931, with an average air temperature 1.04 °C higher than the 1981–2010 baseline. The summer of 2022 reached record temperatures of 47 °C in some regions, representing a critical factor in the increased prevalence and activity of ticks [16]. These vectors are responsible for the dissemination of multiple hemoparasitic agents in animals [17,18].

The One Health concept, which promotes an integrated approach to human, animal, and environmental health, is particularly pertinent in the surveillance of vector-borne infections. Studies focusing on companion animals and the ticks that parasitize them provide a fundamental perspective for understanding the risks affecting both animals and humans, supporting the development of more effective preventive strategies [19].

This study aims to detect infections by hemoparasites in domestic animals at the Hospital Veterinário de Santarém (HVS) and assess the circulation of their tick-borne pathogens in the Santarém region. The findings are correlated with data from the national tick surveillance program (REVIVE-INSA) [20], with a particular focus on their zoonotic potential and the public health implications within a One Health perspective [20]. 

## 2. Materials and Methods

### 2.1. Clinical Data Collected from the Veterinary Hospital

Medical records from 1169 companion animals (823 dogs and 346 cats) treated at the HVS from January 2015 to December 2024 were reviewed. The animals included in the study were residents of the Santarém district and presented with clinically suspected animals of infection by hemoparasites.

**Figure 1:**
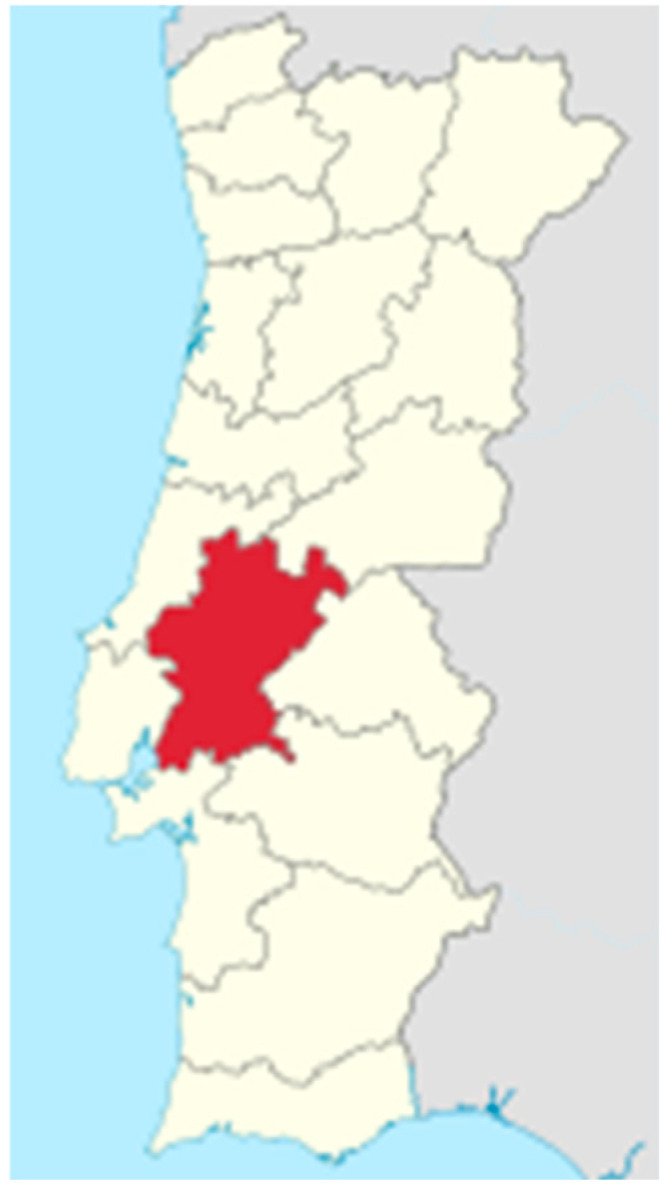
District of Santarém (Portugal). Located in the Lisboa and Vale do Tejo region, it spans an area of 6747 km^2^ and has a population density of approximately 64 inhabitants per km^2^.

The inclusion criteria for this study were as follows:

- Dogs and cats with complete clinical records (breed, age, sex, clinical signs, diagnostic tests performed, test results, treatment administered, history of tick exposure and ectoparasite prevention);
- Residence in the Ribatejo region (District of Santarém) (Figure 1).

- Clinical signs compatible with tick-borne disease (e.g., fever, lethargy, lymphadenopathy, mucosal pallor, petechiae, anemia, thrombocytopenia);
- Serological testing performed for *R. conorii*, *B. canis*, *Erlichia* spp., and/or *M. haemocanis*/*M. haemofelis* (previously referred to as *Haemobartonella* spp.);
- Seropositivity: animals testing positive for at least one of the most common tick-borne pathogens transmitted by ixodid vectors (*R. conorii*, *B. canis*, *Erlichia* spp., and *M. canis*/*M. haemofelis*).

### 2.2. Laboratory Analysis

Blood samples were collected from dogs and cats via jugular or cephalic venipuncture. Blood samples were centrifuged at 3000 rpm for 10 min to obtain serum, which was then stored at −20 °C until testing. Serum samples were analyzed for the presence of antibodies against *R. conorii*, *B. canis*, *Ehrlichia* spp., and *Mycoplasma* spp. using Indirect Immunofluorescence Assay (IFA). Commercially prepared IFA kits provided by DNAtech (Lisbon, Portugal) slides containing fixed antigens of the target hemoparasites were used, with wells specific to each pathogen (e.g., *Ehrlichia* spp., *B. canis*). Serum samples were initially diluted 1:40 in phosphate-buffered saline (PBS, pH 7.2), and in positive or borderline cases, serial two-fold dilutions (1:80, 1:160, 1:320) were performed to determine antibody titers. Next, 25 µL of diluted serum was applied to each well, and the slides were incubated in a humid chamber at 37 °C for 30 min. After incubation, slides were gently washed three times with PBS for 5 min each to remove unbound antibodies. A fluorescein isothiocyanate (FITC)-conjugated anti-dog or anti-cat IgG secondary antibody was then applied, followed by a second 30 min incubation at 37 °C in a humid chamber. The slides were washed again three times with PBS, rinsed with distilled water, and air-dried before mounting medium and coverslips were applied. Slides were examined under an epifluorescence microscope at 400× magnification; positive results were indicated by specific apple-green fluorescence within parasite structures. Antibody titers ≥ 1:80 were considered indicative of prior exposure. Each run included positive and negative control sera supplied with the IFA kit to validate the results.

## 3. Results

Over the past ten years (2015–2024), a total of 3791 serological tests were conducted to investigate suspected infections caused by vector-borne hemoparasites in companion animals treated at the HVS. Serological tests (3.2 tests per animal) were performed on 1169 clinically suspected animals to detect antibodies against *R. conorii*, *B. canis*, *E. canis*, and *Haemobartonella* spp. Of these 1169 animals, 437 tested positive for at least one of the four hemoparasites under investigation. Co-infection was observed in 27% of the animals studied, with *Rickettsia conorii* consistently present in all co-infected cases. The most frequent co-infection involved *R. conorii* and *Haemobartonella* spp., occurring in both dogs and cats. A total of 316 animals were co-infected, including 119 dogs and 197 cats.

The number of tests conducted between 2020 and 2024 nearly tripled compared to the previous five-year period, increasing from 894 to 2883. This was accompanied by a rise in positive cases and an increase in average prevalence, from 29.5% to 39.9% (Table 1 and Table 2).

*R. conorii* showed the highest seroprevalence in companion animals in the Ribatejo region from 2015 to 2019, with 174 positive cases (prevalence of 60.2%), followed by *Haemobartonella* spp. with 34 positive cases (prevalence rate of 54.8%). However, from 2019 to 2024, *Haemobartonella* spp. reached a prevalence similar to *R. conorii*, accounting for approximately 66% of cases, with 458 positive cases, 197 of them detected in cats. *R. conorii* showed an average prevalence of 64.8% during the study period, with higher rates in dogs (67.0%) compared to cats (58.6%). In both species, this suggests significant exposure to the vector *R. sanguineus*, which is considered the main transmission vector.

*B. canis* infection was exclusively detected in dogs, with a total of 84 confirmed cases (prevalence of 10.4%) over the study period. The absence of positive cases in cats aligns with the agent’s life cycle and its preferential hosts, which primarily include dogs and the tick vector *D. reticulatus.*

Regarding *Ehrlichia* spp. infections, 115 positive cases were recorded, 57 in dogs and 68 in cats, with a prevalence of 6.9% and 23.1%, respectively, and an overall prevalence of 10.4%. In contrast, *Haemobartonella* spp. (now classified under the genus *Mycoplasma*) was significantly more frequent in cats (223 positive cases) than in dogs (269 positive cases), with respective prevalence rates of 87.5% and 53.37%, and an overall prevalence of 66.4%.

## 4. Discussion

In the Ribatejo region, data from the HVS indicate a high prevalence of *R. conorii* (64.8%) with higher rates in dogs (67.0%) compared to cats (58.6%). This reflects the abundance of *R. sanguineus*, the primary vector of this hemoparasitosis in the area. Both animal species played a relevant role in the surveillance of *R. conorii*. During 2015–2019, 174 positive cases of *R. conorii* were recorded, increasing to 542 in 2020–2024, indicating intensified circulation of its tick vector. The post-2020 rise in *R. conorii* infections in companion animals may reflect increased exposure or improved diagnostic efforts. The predominance of *R. sanguineus* was also evidenced by the results of the REVIVE program (Appendix B adapted from [20]). Moreover, the detection of co-infection in 27% of the animals studied, with *R. conorii* consistently present in all such cases, strongly underscores the central role of this pathogen in the epidemiological landscape of vector-borne infections in companion animals. The predominance of co-infection with *Haemobartonella* spp. in both dogs and cats suggests a convergent transmission pathway, likely mediated by a common ectoparasitic vector, such as *R. sanguineus* (Table 1 and Table 2). This vector is well-documented for its broad host range, endophilic behavior, and capacity to harbor and transmit multiple pathogens. Its involvement in both canine and feline infections indicates a shared ecological niche and facilitates cross-species transmission, particularly in cohabiting domestic environments [4,5,6].

The REVIVE Program (Rede de Vigilância de Vetores), coordinated by the Instituto Nacional de Saúde Doutor Ricardo Jorge (INSA) includes available reports with information on tick species distribution, host preferences, and pathogen detection in Portugal [20]. According to data from the REVIVE program, the highest number of tick collections was recorded in 2023, with over 1600 samples. This increase reflects a growing presence and circulation of tick vectors in Portugal (Appendix A adapted from [20]). In 2023, six Rickettsia species were detected in collected ticks, with an average prevalence of 25.1%, particularly in specimens removed from humans. The total number of ixodids collected directly from hosts showed a fluctuating trend. From 2015 to 2018, there was a significant increase, peaking in 2018 with 568 ticks. This was followed by a steady decline between 2019 and 2022, reaching the lowest point in 2022 with 243 ticks. In 2023 the numbers rose again, with 430 ticks collected. Among the tick species monitored, *R. sanguineus* consistently showed the highest relative abundance across all years, ranging from 57% to 82%. In 2020, its prevalence dropped significantly to 57.4%, followed by a slight recovery in subsequent years. *I. ricinus* demonstrated a steady increase in prevalence, rising from 3.5% in 2014 to 10.5% in 2023 [20] *D. marginatus* maintained a relatively stable presence, with a notable increase in 2020 (7%) and again in 2021 (6.6%). *H. lusitanicum* exhibited an increasing trend from 1% in 2015 to 3.7% in 2022, followed by a drop to 0.7% in 2023. Other species, such as *H. punctata*, *I. hexagonus*, *I. ventalloi*, *I. frontalis*, *D. reticulatus*, and *R. pusillus*, showed consistently low relative abundance (adapted from [20]).

Serological tests at HVS also confirmed the presence of the hemoparasite *B. canis* in dogs, with a prevalence of 13% between 2015 and 2019, and 8.8% between 2020 and 2024. These findings highlight the central role of dogs in the epidemiological surveillance of *B. canis*, which is transmitted by *D. reticulatus.* The absence of positive cases of *Babesia* in cats aligns with the agent’s life cycle and its preferential hosts, which primarily include dogs and the vector *D. reticulatus*. These results underscore the distinct roles that dogs and cats play as hosts in the transmission of tick-borne infections.

*Haemobartonella* spp. (now reclassified under the genus *Mycoplasma*) was found to be significantly common in cats, with 223 positive cases compared to 269 positive cases in dogs. The respective prevalence rates were 87.5% in cats and 53.37% in dogs, resulting in an overall prevalence of 66.4%. This pattern aligns with current knowledge on *M. haemofelis* epidemiology, a pathogen commonly found in cats. The primary vector is the flea *Ctenocephalides felis*, although transmission via *R. sanguineus* is also possible (Table 1 and Table 2). Infections by *Ehrlichia* spp. showed a prevalence of 6.9% in dogs and 23.1% in cats, with an overall average of 10.4%. Although dogs are generally more susceptible, particularly to *E. canis*, transmitted by *R. sanguineus*, the notable prevalence in cats may reflect their frequent outdoor exposure in rural areas of the Ribatejo region.

Hemoparasitic infections in dogs and cats often present with nonspecific clinical signs such as fever, lethargy, lymphadenopathy, and anemia, often making them difficult to distinguish without laboratory testing. Co-infections can result in serious complications, including renal failure, disseminated intravascular coagulation (DIC), and encephalopathy, further complicating both diagnosis and treatment [21,22]. The overlap of different vector species and circulating agents promotes co-infections, which are associated with more severe clinical outcomes, especially in immunocompromised animals [21,23]. *R. conorii* is by far the most prevalent vector-borne agent identified in Portugal, as evidenced by results from present study which is in line with REVIVE data [20]. Analysis of the decade-long dataset reveals distinct positivity patterns between dogs and cats, reflecting differences not only in species susceptibility but also in vector exposure. The tick species of greatest epidemiological relevance are *R. sanguineus*, *I. ricinus*, *D. marginatus*, *D. reticulatus*, *H. lusitanicum*, and *H. marginatum* [24]. These species have the potential to transmit various zoonotic Mediterranean spotted fever (also known as boutonneuse fever) in humans, a serious disease that can progress to multiorgan complications [16,25]. While *R. sanguineus* is the primary vector, *Ixodes* spp., *Dermacentor* spp., and *Hyalomma* spp. can also act as secondary vectors [14,26]. *I. ricinus* is particularly concerning due to its wide geographic distribution and its capacity to transmit a diverse range of pathogens, including *Borrelia burgdorferi* s.l. (the causative agent of Lyme borreliosis), *Anaplasma phagocytophilum*, *Francisella tularensis*, and *Rickettsia helvetica* [9,17]. Moreover, *I. ricinus* is widely distributed in Europe and is the main vector of TBEV an emerging viral zoonosis [10]. The *Dermacentor* genus, especially *D. reticulatus* and *D. marginatus*, is associated with *B. canis* and *Rickettsia slovaca*, respectively, both causing clinical conditions such as TIBOLA (tick-borne lymphadenopathy), particularly in vulnerable groups [10]. *Hyalomma* spp. are notable vectors of CCHF and *Rickettsia aeschlimannii*, both of which have significant clinical implications [11,12].

Two out of the four haemoparasites discussed in this study have known zoonotic potential namely *R. conorii* and *Ehrlichia* spp; however, the possibility of zoonotic spillover of agents such as *Babesia* spp. and hemoplasmas (e.g., *Mycoplasma haemocanis* or *M. suis*). Documented cases of *Babesia divergens* in patients in Europe, including immunocompetent individuals, have been confirmed by PCR and 18S rDNA sequencing, demonstrating the ability of this protozoan to overcome interspecific barriers [13]. With regard to hemoplasmas, there have been reports of human infection by a *Mycoplasma haemofelis*-like organism in an HIV-positive patient [15]. Climatic conditions influence the host-seeking behavior of *R. sanguineus*, with higher temperatures increasing both tick aggressiveness and biting frequency, thereby raising the risk of human infection [18,27,28]. Furthermore, the summer season coincides with increased outdoor recreational activities, which heightens the likelihood of human contact with questing ticks. After a tick bite, *R. conorii* typically requires between 3 and 24 h to be effectively transmitted. Clinical signs spotted fever group rickettsioses include high fever, flu-like symptoms, prostration, a maculopapular or petechial rash, and a characteristic black eschar at the site of the tick bite. In severe cases, neurological complications such as encephalitis may occur, particularly in elderly or immunocompromised individuals, or in those with comorbidities [18]. Phylogenetic analyses based on complete genome sequencing have shown that the distribution of spotted fever group rickettsioses species is closely linked to the presence of specific tick vectors and the population dynamics of their vertebrate hosts, highlighting the importance of vector presence for disease circulation [18,29]. Although spotted fever group rickettsioses have a global distribution, most reported human cases have occurred in Europe and the United States, and surveillance remains insufficient in many regions [29].

Also transmitted by *R. sanguineus*, *E. canis* is the etiological agent of canine monocytic ehrlichiosis (CME), an infectious disease with recognized zoonotic potential. Although classically considered to be restricted to dogs, sporadic cases in humans have been reported [30,31,32]. Human infection can manifest with non-specific symptoms such as fever, headache, asthenia and hematological changes, and is often underdiagnosed. The species most frequently associated with human monocytic ehrlichiosis (HME) is *Ehrlichia chaffeensis*, endemic to the United States [1] which can also be identified in dogs, although rarely [2]. This tick-borne bacterial human infection remains underreported worldwide, with most available data coming from case reports, case series, and retrospective studies; prospective studies and clinical trials are still scarce [1]. Clinically, HME presents as a non-specific febrile illness (95% of cases), frequently accompanied by thrombocytopenia (79.1%), leukopenia (57.8%), and abnormal liver function tests (68.1%) [1]. The serological distinction between *E. chaffeensis* and *E. canis* is difficult due to the cross-reactivity between the two species [33], thereby complicating accurate species identification and epidemiological surveillance. Although human infections with *Ehrlichia* species other than *E. chaffeensis* remain rare, the emerging zoonotic potential of *E. canis*, its shared vector with other zoonotic agents and the diagnostic challenges associated with serological cross-reactivity reinforce the need for enhanced surveillance and the use of molecular diagnostic techniques in endemic areas [32]. In recent years, the global emergence and re-emergence of VBDs has intensified. This dynamic is driven by factors such as globalization, movement of goods, climate change, and an increasing number of susceptible hosts [22,34]. Climate change, especially rising average temperatures, directly affects vector ecology by extending both their seasonal activity and geographic range. Portugal is considered increasingly at risk, due to the established presence of competent vectors [35,36,37]. Moreover, the District of Santarém, located 20 to 50 km from the Atlantic coast, is well connected through seaports, regional airports, and a dense road network. This strong infrastructure supports trade, tourism, and economic activities such as agriculture, manufacturing, and commerce. However, such connectivity also increases the risk of TBD introduction, as vectors may arrive via planes, ships, or migratory birds, underscoring the importance of vector monitoring. Data from the REVIVE Program (2011–2023) confirm the presence of *Hyalomma*, *Dermacentor*, *Rhipicephalus*, and *Ixodes* throughout the country, justifying concern over the potential emergence or reemergence of TBD.

The data obtained in this study, comparing regional veterinary findings with national REVIVE Program data, points to a high overlap of vector species with zoonotic potential. The presence of pathogens in companion animals, often asymptomatic, represents an indirect risk to human health [38,39]. This work reinforces the importance of integrated One Health approaches for the surveillance and control of vectors and emerging diseases [37]. Ongoing monitoring, early diagnosis, and cooperation among human, veterinary, and environmental medicine are essential to anticipate and mitigate the growing risk of TBD in Portugal [40]. These findings highlight the need for integrated surveillance and a One Health approach to clarify the transmission of zoonotic hemoparasites, improve diagnostic accuracy, and enhance preparedness for emerging threats. The consistent co-detection of *Rickettsia conorii* with other hemotropic pathogens suggests possible vector co-infection or sequential transmission, which may support pathogen persistence and increase the risk of zoonotic spillover.

## 5. Conclusions

The high prevalence of *Rickettsia conorii* (64.8%) underscores the epidemiological importance of *Rhipicephalus sanguineus* as a primary vector in the Ribatejo district. Dogs were key hosts for *Babesia canis* and *Ehrlichia canis*, while cats were mainly affected by *Haemobartonella* spp. (*Mycoplasma* spp.). These host–pathogen patterns should guide surveillance and control efforts. The presence of zoonotic agents like *R. conorii* and *Ehrlichia* highlights a potential risk to human health, reinforcing the need for a One Health approach.

## Figures and Tables

**Table 1:** Serological tests performed between 2015 and 2019 in dogs and cats with suspected hemoparasitosis.

Parasite (Primary Vector)	Serologic Tests (No)	Dogs		Cats		Total	
		Negative (No)	Positive (No)	Negative (No)	Positive (No)	Negative (No, %)	Positive (No, %)
*Rickettsia conorii* *(Rhipicephalus sanguineus)*	289	99	157 ^a^	16	17	115 (39.8)	174 (60.2)
*Babesia canis* *(Dermacentor reticulatus)*	253	220	18 + 15 ^b^	0	0	220 (87.0)	33 (13.0)
*Ehrlichia* spp. *(Riphicephalus sanguineus)*	290	240	17	27	6	267 (92.0)	23 (7.9)
*Haemobartonella* spp. *(Ctenocephalides felis* *Riphicephalus sanguineus)*	62	21	8	7	26	28 (45.2)	34 (54.8)

^a^ titles of 1/40 and 1/180; ^b^ basal fluorescence. No = number.

**Table 2:** Serological tests performed between 2020 and 2024 in dogs and cats with suspected hemoparasitosis.

Parasite (Primary Vector)	Serologic Tests (No)	Dogs		Cats		Total	
		Negative (No)	Positive (No)	Negative (No)	Positive (No)	Negative (No, %)	Positive (No, %)
*Rickettsia conorii* *(Rhipicephalus sanguineus)*	816	168	386 ^a^	106	156	274 (33.6)	542 (66.4)
*Babesia canis* *(Dermacentor reticulatus)*	554	503	16 + 35 ^b^	0	0	503 (91.2)	51 (8.8)
*Ehrlichia* spp. *(Riphicephalus sanguineus)*	830	529	37 + 3 ^b^	199	62	728 (88.8)	92 (11.2)
*Haemobartonella* spp. *(Ctenocephalides felis* *Riphicephalus sanguineus)*	697	214	261	25	197	239 (34.5)	458 (65.7)

^a^ titles of 1/40 and 1/180; ^b^ basal fluorescence. No = number.

**Figure A1:**
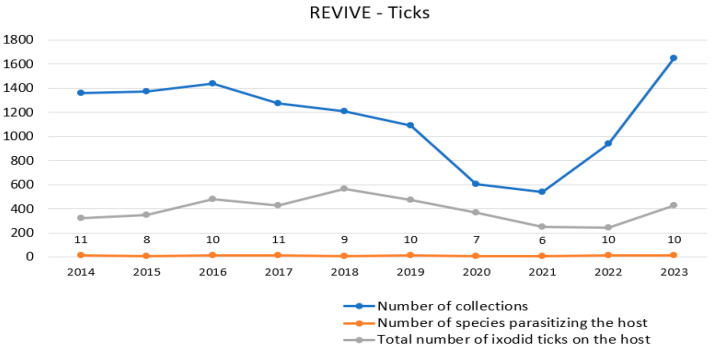
REVIVE data from samples from the last 10 years (2014 to 2023) [20].

## Data Availability

The original contributions presented in this study are included in the article. Further inquiries can be directed to the corresponding author.

## Abbreviations

The following abbreviations are used in this manuscript:

TBD	Tick-borne Diseases
VBD	Vector-borne Diseases
IFA	Indirect Immunofluorescence Assay
DIC	Disseminated Intravascular Coagulation
CCHFV	Crimean Congo Hemorrhagic Fever Virus
DIC	Disseminate intravascular coagulation
HVS	Hospital Veterinário de Santarém
INSA	Instituto Nacional de Saúde Doutor Ricardo Jorge
PCR	Polymerase Chain Reaction
TBEV	Tick-borne encephalitis virus
REVIVE	Rede de Vigilância de Vetores
CME	Canine monocytic ehrlichiosis
HME	Human monocytic ehrlichiosis

## Appendix A

Figure A1 presents data from the REVIVE-INSA Program over the past ten years (2014–2023), providing insights into tick surveillance in Portugal. This report summarizes the number of tick collections, the number of tick species parasitizing hosts, and the total number of ixodid ticks identified on hosts each year.

The diversity of tick species parasitizing hosts fluctuated annually. The highest recorded diversity occurred in 2017, with 11 species identified, indicating a high level of ecological variation. This was followed by a notable decline in 2020 and 2021, with only 7 and 6 species reported, respectively, the lowest counts in the time series. Species diversity recovered in 2022 and 2023, reaching ten species in both years.

## Appendix B

Pathogenic agents detected to date (2011–2023) by Polymerase Chain Reaction (PCR) and their respective vectors (adapted from REVIVE 2023) in Portugal are presented in the table above.

**Table A1:** Disease-causing pathogens detected to date (2011–2023) by the Polymerase Chain Reaction (PCR) technique and their respective vectors: (Revive 2023, adapted from [20]).

Vector/Agent/Disease	*Ixodes hexaginus*	*Ixodes ricinus*	*Ixodes ventaloi*	*Ixodes frontalis*	*Hyalomma marginatus*	*Hyalomma reticulatus*	*Hyalomma lusitanicum*	*Hyalomma marginatum*	*Dermacentor sanguineus*	*Rhipicephalus pusillus*
Associated Disease(s)	Babesiosis (*); Borreliosis (*); Anaplasmosis	Borreliosis (*); Anaplasmosis; Tularemia (*); TBEV Encephalitis	Anaplasmosis	No Designation	TBEV Encephalitis; Q Fever (*)	TIBOLA (*)	Q Fever (*); Tularemia (*); TIBOLA (*)	CCHF (*); Q Fever (*); LAR (*)	CCHF (*)	Nodular Fever (*); LAR (*)
2014	<1	3.2	<1	0	<1	2.4	1.6	1.4	1.2	88.1
2015	<1	3.5	<1	0	<1	4.3	1	1	2.4	82.2
2016	<1	5	<1	0	<1	4.7	<1	<1	2.4	81.4
2017	0	5.4	<1	<1	<1	3.4	1.4	1.7	1.7	78
2018	<1	6.5	<1	<1	0	2	1.6	2.4	3.4	80.5
2019	<1	7.9	<1	<1	<1	1.7	0.8	2.1	3.7	75.2
2020	<1	20.3	<1	<1	0	7	1	2.1	7	57.4
2021	<1	7.2	<1	0	0	6.6	1.8	1.2	1.8	79.9
2022	1–3	8.6	<1	0	<1	6.3	0.3	3.7	4.3	71
2023	1.2	10.5	0.1	0	0.6	4.2	1	0.7	2	75.6

***** Already described in Portugal.
